# Noninvasive rapid urinary trypsinogen‐2 dipstick test for early exclusion of post‐endoscopic retrograde cholangiopancreatography pancreatitis within hours after endoscopic retrograde cholangiopancreatography: Clinical diagnosis and considerations

**DOI:** 10.1002/deo2.336

**Published:** 2024-02-21

**Authors:** Kazuki Hama, Atsushi Sofuni, Ryosuke Nakatsubo, Takayoshi Tsuchiya, Reina Tanaka, Ryosuke Tonozuka, Shuntaro Mukai, Kenjiro Yamamoto, Akio Katanuma, Takao Itoi

**Affiliations:** ^1^ Department of Gastroenterology and Hepatology Tokyo Medical University Tokyo Japan; ^2^ Center for Gastroenterology Teine Keijinkai Hospital Hokkaido Japan

**Keywords:** diagnosis, endoscopic retrograde cholangiopancreatography, post‐ERCP pancreatitis, rapid urinary trypsinogen‐2 dipstick test, trypsinogen‐2

## Abstract

**Objective:**

Few reports have explored the application of urinary trypsinogen‐2 measurement in the early diagnosis of post‐endoscopic retrograde cholangiopancreatography (ERCP) pancreatitis, and none have demonstrated the benefits of noninvasive testing. This study aimed to evaluate the clinical application of the rapid urinary trypsinogen‐2 dipstick test (Nipro, Japan) compared with serum amylase and lipase levels for the early diagnosis of post‐ERCP pancreatitis (PEP).

**Methods:**

A total of 100 consecutive patients (54 men and 46 women) who were admitted and underwent ERCP at Tokyo Medical University Hospital from August 2021 to December 2021 were recruited. All patients underwent rapid urinary trypsinogen‐2 measurement using the dipstick test before and after ERCP. Measurements were taken 24 h before ERCP for pre‐ERCP and 1–4 h after ERCP for post‐ERCP. Additionally, serum amylase and lipase levels were measured at 8:00 a.m. of the day after ERCP (at least 8 h after ERCP), and their diagnostic abilities for PEP were compared and evaluated.

**Results:**

PEP occurred in 5/100 patients (5%). The sensitivity, specificity, positive predictive value, and negative predictive value of the dipstick test for diagnosing PEP were 100%, 83.2%, 23.8%, and 100%, respectively. These results were comparable to the diagnostic performance of serum amylase and lipase levels at 8:00 a.m. on the day after ERCP (at least 8 h after ERCP). However, false positives must be considered.

**Conclusion:**

The dipstick test may be useful in clinical practice as a noninvasive screening test for the early prediction of PEP.

## INTRODUCTION

Endoscopic retrograde cholangiopancreatography (ERCP) is an important procedure for the treatment of biliary pancreatic diseases. Currently, endoscopic ultrasound (EUS) and EUS‐guided fine‐needle aspiration are the mainstream methods for the diagnosis of pancreatic masses and cystic lesions; however, ERCP is still an essential test method when a mass cannot be identified or when there is an abnormality in the pancreatic duct.

Complications of ERCP include post‐ERCP pancreatitis (PEP), bleeding, perforation, and infection. Of these, PEP is the most serious and potentially fatal. A survey of 21 studies targeting 16,855 patients indicated that the incidence and mortality rates of ERCP‐related complications were 6.85% and 0.33%, respectively, among which PEP was one of the major complications. PEP occurs in approximately 1%–10% of patients who undergo ERCP and is reported to be mild to moderate in the majority of patients; however, some patients develop severe pancreatitis, causing a mortality rate of 0.11%.^1^

Serum amylase and lipase levels are used in PEP diagnosis; however, it is known that in approximately 25%–75% of patients, stimulation of the bile and pancreatic ducts increases serum amylase and lipase levels independently of PEP.^2^

According to the guidelines of the European Society of Gastrointestinal Endoscopy (ESGE), PEP was defined as new or worsening abdominal pain lasting at least 24 h after ERCP, serum amylase or lipase levels exceeding three times the upper reference limit, and radiographs consistent with pancreatitis (all within 3 days after ERCP).^3^ However, the presence of abdominal pain that persists for 24 h requires immediate action. Therefore, the diagnosis of PEP is mostly based on abdominal symptoms on the day after ERCP or by computed tomography tests.

Measurement of urinary trypsinogen‐2 was made possible in the 1990s. It has been reported that urinary trypsinogen‐2 measurements are useful for the diagnosis of acute pancreatitis. However, these measurements remain limited, and there are few reports on whether they are useful for PEP diagnosis and, by extension, early diagnosis of PEP within hours after ERCP.

This study aimed to evaluate the clinical application of the rapid urinary trypsinogen‐2 dipstick test (Nipro, Japan) for the early diagnosis of PEP compared to serum amylase and lipase levels.

## MATERIALS AND METHODS

### Patients

**FIGURE 1:**
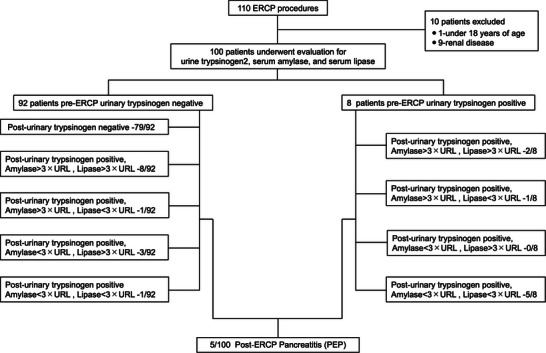
The participants of this study were patients who underwent ERCP at the Department of Gastroenterology and Hepatology at Tokyo Medical University Hospital from August 2021 to December 2021, with a total of 100 patients (54 men and 46 women) eventually included. Of the 100 patients, five patients (5%) had PEP. All five patients with PEP tested positive with the rapid urinary trypsinogen‐2 dipstick test. ERCP, endoscopic retrograde cholangiopancreatography; PEP, post‐endoscopic retrograde cholangiopancreatography pancreatitis.

This study included 100 consecutive patients (54 men and 46 women) who were admitted and underwent ERCP at the Department of Gastroenterology and Hepatology at Tokyo Medical University Hospital between August and December 2021 (Figure 1). The exclusion criteria were as follows: 1) patients who developed PEP within the last 60 days; 2) patients under 18 years of age; 3) pregnant patients; 4) patients with known renal disease; and 5) patients who underwent hepaticojejunostomy.

The study protocol was approved by the Ethics Committee of Tokyo Medical University Hospital (T2022‐0071).

### Endoscopic procedures

ERCP was conducted by an experienced gastroenterologist (a physician with at least 7 years total and 4 years ERCP experience) using the Olympus TJF260V or SIF‐H290S (Olympus Medical). Sedation was administered transvenously, using flunitrazepam or dexmedetomidine hydrochloride. A breakdown of the ERCP procedures is as follows: endoscopic sphincterotomy, endoscopic papillary balloon dilation, endoscopic papillary large balloon dilation, endoscopic pancreatic sphincterotomy, endoscopic papillectomy (EP), peroral cholangioscopy, intraductal ultrasonography, stone removal (basket catheter, balloon catheter, and endoscopic mechanical lithotripsy), endoscopic biliary stent, and endoscopic pancreatic duct stent. These procedures were performed according to ERCP indications. Spontaneous pancreatic duct plastic stent placement was performed if the pancreatic duct was accidentally cannulated more than once during the ERCP. The endoscopic pancreatic duct stent (with the flap) was placed in all cases where the EP was conducted.

### Methods

All the patients underwent a rapid urinary trypsinogen‐2 dipstick test before and after ERCP. The measurement times were 24 h before ERCP for pre‐ERCP and 1–4 h after ERCP for post‐ERCP, considering the individual differences in urination associated with treatment. Additionally, serum amylase and lipase levels were measured at 8:00 a.m. on the day after ERCP (at least 8 h after ERCP). After ERCP, 50 mg of diclofenac sodium was administered anally to prevent pancreatitis. During the observation period, the clinical course of all patients was monitored until the patient was discharged from the hospital or died.

### Rapid urinary trypsinogen‐2 dipstick test

**FIGURE 2:**
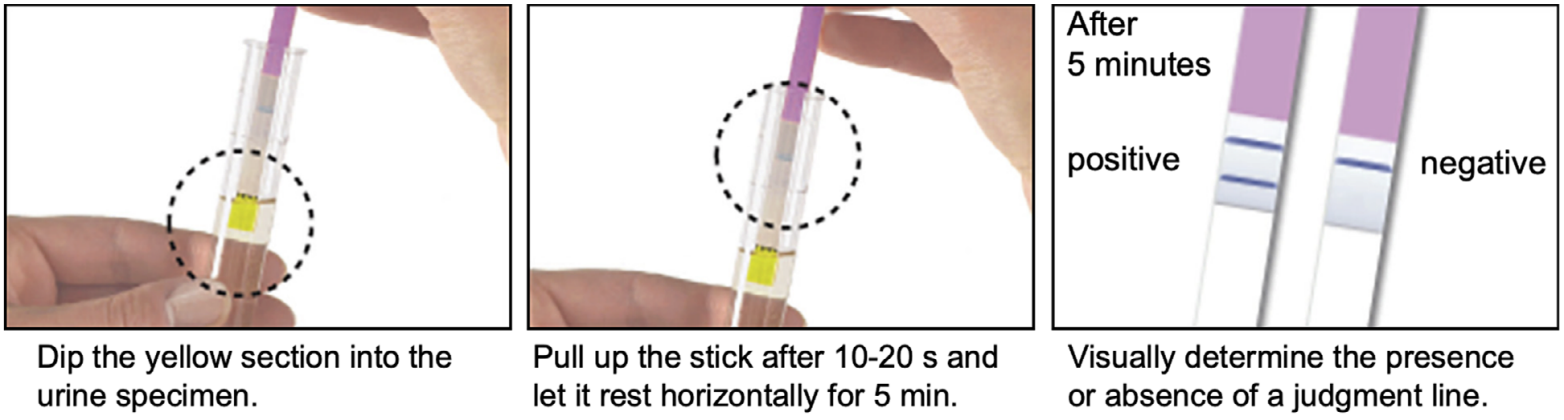
The results were judged as positive when two blue lines appeared.

The rapid urinary trypsinogen‐2 dipstick test is based on immunochromatography. The tip of the dipstick was immersed in the urine specimen and allowed to stand for 5 min. If the urinary trypsinogen‐2 concentration was excessive (50 μg/L or more), two blue lines were observed (one denoting trypsinogen‐2 and the other denoting the control); if the urinary trypsinogen‐2 concentration was within the normal range, only one blue line (control) was observed. Results were considered positive when two blue lines appeared (Figure 2). If no control line was detected, another rapid dipstick test was performed on the urine specimen.

### Definitions

The definition of PEP was evaluated according to the guidelines of the ESGE. PEP was diagnosed when two of the following were present: new or worsening abdominal pain lasting at least 24 h after ERCP, serum amylase or lipase levels exceeding three times the upper reference limit, and radiographs consistent with pancreatitis (all within 3 days after ERCP).

For the severity of pancreatitis, the severity classification by Cotton et al.^4^ was applied, with mild, moderate, and severe classifications applied for hospitalization extensions of 2–3, 4–10, and >10 days, respectively. Severe classifications were also used if pancreatic necrosis, abscess, or pseudocysts developed, or if percutaneous drainage or surgery was required.

Patients who tested positive with the rapid trypsinogen‐2 dipstick test after ERCP but did not meet the diagnostic criteria for PEP were defined as false positives.

Serum amylase levels were measured using an automatic clinical chemistry analyzer (Labospect008 Hitachi LABOSPECT 008; Hitachi High‐Tech Co.). Serum lipase levels were measured using an automatic biochemical analyzer, Roche cobas8000 (Roche Diagnostics). The standard values were 39–124 U/L for amylase and 13–49 U/L for lipase.

Patients who did not meet the diagnostic criteria for PEP were diagnosed with hyperamylasemia if their amylase levels exceeded the upper reference limit.

### Outcomes

The primary endpoint was to assess the ability of the rapid urinary trypsinogen‐2 dipstick test to diagnose PEP compared to serum amylase and lipase levels. The secondary endpoints included assessing serum amylase and lipase levels in false‐positive patients and evaluating the clinical application of the rapid urinary trypsinogen‐2 dipstick test.

### Statistical analysis

Fisher's exact test was used to compare categorical data. *p*‐values <0.05 were considered statistically significant. For positive predictive value (PPV), differences and 95% confidence intervals were calculated. EZR^5^ was used for all the statistical analyses. EZR is a statistical software that extends the functionality of R and R Commander.

## RESULTS

A total of 100 patients (54 men and 46 women) with a median age of 70.2 years (range, 18–97 years) were recruited.

Table 1 shows the characteristics of the 100 patients who underwent ERCP, along with a breakdown of diagnosis and treatment. Common bile duct stones were the most common diagnosis. Those who underwent endoscopic sphincterotomy totaled 35 patients (three of whom had PEP); endoscopic papillary large balloon dliation, eight patients (two with PEP); endoscopic papillary balloon dilation, five patients; endoscopic pancreatic sphincterotomy, five patients; EP, 12 patients (one with PEP); peroral cholangioscopy, eight patients; and intraductal ultrasonography, three patients. The median ERCP treatment time was 28 min (range, 4–131 min).

**TABLE 1:** Patient characteristics.

	No. of patients (*n* = 100)	No. of patients with post‐ERCP pancreatitis (*n* = 5)
**Age, median (range), years**	72 (18–97)	63 (52–79)
**Sex (male/female), *n***	54/41	3/2
**Diagnosis, *n* (%)**		
Common bile duct stone	39 (39%)	1 (20%)
Papillary adenoma	12 (12%)	2 (40%)
Chronic pancreatitis	12 (12%)	N/A
Pancreatic cancer	10 (10%)	1 (20%)
Cholangiocarcinoma	8 (8%)	1 (20%)
Intraductal papillary mucinous neoplasm	3 (3%)	N/A
Sphincter of Oddi dysfunction	2 (2%)	N/A
Intrahepatic stones	2 (2%)	N/A
Benign pancreatic duct stricture	2 (2%)	N/A
Malignant bile duct stricture	2 (2%)	N/A
Autoimmune pancreatitis	2 (2%)	N/A
Postoperative bile duct stenosis	2 (2%)	N/A
Pancreas divisum	1 (1%)	N/A
Benign bile duct stricture	1 (1%)	N/A
Intraductal papillary neoplasm of bile duct	1 (1%)	N/A
Gallbladder cancer	1 (1%)	N/A
**Treatment details**		
Procedure time, median (range), minutes	28 (4–131)	29 (27–63)
Naïve papilla, *n* (%)	59 (59%)	4 (80%)
Endoscopic sphincterotomy, *n* (%)	35 (35%)	3 (60%)
Endoscopic papillary balloon dilation, *n* (%)	5 (5%)	N/A
Endoscopic papillary large balloon dilation, *n* (%)	6 (6%)	2 (%)
Endoscopic papillectomy, *n* (%)	12 (12%)	1 (20%)
Endoscopic pancreatic sphincterotomy, *n* (%)	5 (5%)	N/A
Peroral cholangioscopy, *n* (%)	8 (8%)	N/A
Intraductal ultrasonography, *n* (%)	3 (3%)	N/A
Endoscopic biliary stent, *n* (%)	43 (43%)	4 (80%)
Endoscopic pancreatic duct stent, *n* (%)	19 (19%)	2 (40%)

Of the 100 patients, five patients (5%) had PEP. All five patients had mild disease, and none had severe disease. The median ERCP treatment duration for patients who developed PEP was 29 min (range, 27–63 min). There were no significant differences in treatment time (*p* = 0.63). All five patients with PEP tested positive in the rapid urinary trypsinogen‐2 dipstick test. Of the five patients, two underwent rapid tests 1 h after ERCP, one underwent rapid tests 2 h after ERCP, and two underwent rapid tests 4 h after ERCP due to individual differences in urination. Although none of the five patients complained of abdominal pain at the time of the urinary trypsinogen‐2 rapid measurement, they complained of abdominal pain at 8:00 on the day after ERCP (at least 8 h after ERCP).

Table 2 compares the sensitivity, specificity, PPV, and negative predictive value (NPV) of the rapid urinary trypsinogen‐2 test for serum amylase and lipase levels. The sensitivity, specificity, PPV, and NPV of the rapid urinary trypsinogen‐2 test for PEP diagnosis were 100%, 83.2%, 23.8%, and 100%, respectively. For the PEP diagnostic ability of serum amylase and lipase levels at 8:00 a.m. on the day after ERCP (at least 8 h after ERCP), the PPV of serum amylase at a cutoff level of three times the upper reference limit was 20.8%. The PPV of serum lipase at a cutoff level three times the upper reference limit was 20.0%.

**TABLE 2:** Comparison of the urinary trypsinogen‐2 test, serum amylase, and lipase levels for the detection of post‐endoscopic retrograde cholangiopancreatography (post‐ERCP) pancreatitis.

	Urinary trypsinogen‐2	Urinary trypsinogen‐2 (excluding pre‐positive)	Amylase >3×URL	Lipase >3×URL
Sensitivity, %	100	100	100	100
Specificity, %	83.2	90.8	80.0	78.9
PPV, %	23.8	38.5	20.8	20.0
NPV, %	100	100	100	100

Abbreviations: ERCP, endoscopic retrograde cholangiopancreatography; NPV, negative predictive value; PPV, positive predictive value; URL, upper reference limit.

Additionally, eight patients (8%) were positive for the rapid urinary trypsinogen‐2 dipstick test before ERCP (24 h prior to the procedure). All eight remained positive after ERCP and did not develop PEP. The sensitivity, specificity, PPV, and NPV of the rapid urinary trypsinogen‐2 dipstick test, excluding these eight patients, were 100%, 90.8%, 38.5%, and 100%, respectively.

**FIGURE 3:**
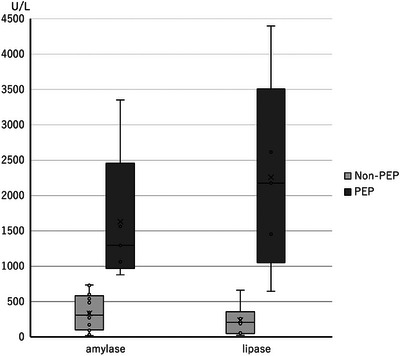
Comparison of serum amylase and lipase levels in 21 patients who tested positive on the rapid urine trypsinogen‐2 dipstick test after ERCP. The median (IQR) of amylase in the non‐PEP patients was 308 (98–585) U/L and that of lipase was 206 (47–358) U/L. The median (IQR) of amylase and lipase in PEP patients were 1295 (970–2458) U/L and 2177 (1049–3507) U/L, respectively. Serum amylase and lipase levels in PEP patients were significantly higher (*p* < 0.05). ERCP, endoscopic retrograde cholangiopancreatography; IQR, interquartile range; PEP, post‐endoscopic retrograde cholangiopancreatography pancreatitis.

There were 21 patients (21%) who tested positive on the rapid urinary trypsinogen‐2 dipstick test after ERCP. Of the 21 rapid urinary trypsinogen‐2 dipstick test‐positive cases after ERCP, five had PEP, and 16 were false positives. Of the false‐positive cases, 10 were diagnosed as hyperamylasemia. Figure 3 shows a comparison of the serum amylase and lipase levels in the 21 positive subjects. Of the 21 subjects, five patients who developed PEP had significantly higher serum amylase and lipase levels than those 16 who did not develop PEP (*p* < 0.001).

**FIGURE 4:**
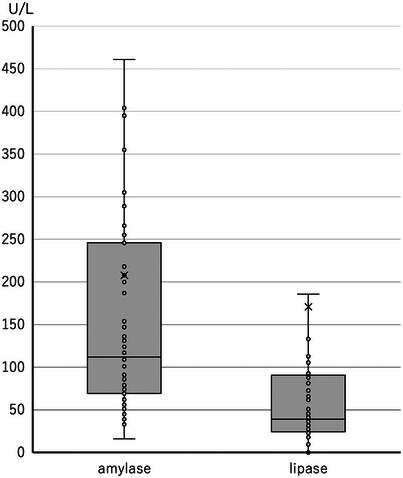
Serum amylase and lipase levels of the 79 patients who tested negative in the rapid urine trypsinogen‐2 dipstick test after endoscopic retrograde cholangiopancreatography. The amylase median (interquartile range) was 112 (69–246) U/L and the lipase median (interquartile range) was 39 (24–90) U/L.

Furthermore, we examined the amylase and lipase levels in 79 patients who tested negative on the rapid urine trypsinogen‐2 dipstick test after ERCP. As illustrated in Figure 4, the serum amylase and lipase levels on the day after ERCP (at least 8 h after ERCP) were significantly lower than those for PEP. Thus, a negative rapid urine trypsinogen‐2 dipstick test may not show serum amylase or lipase levels exceeding three times the upper reference limit, which is the diagnostic criterion for PEP.

## DISCUSSION

This study demonstrated that the rapid urinary trypsinogen‐2 test could exclude the possibility of PEP within hours following ERCP.

Reports have indicated that serum and urinary trypsinogen‐2 levels are elevated in patients with acute pancreatitis and that trypsinogen‐2 levels may be a more sensitive diagnostic marker for pancreatitis than amylase and lipase.^6^, ^7^ Trypsinogen, a precursor of trypsin, is secreted from the acinar cells into the pancreatic juice. The two major isoenzymes of trypsinogen are trypsinogen‐1 and trypsinogen‐2, and intrapancreatic activation of trypsinogen to trypsin is thought to play an important role in the pathogenesis of acute pancreatitis.^8^ In acute pancreatitis, both trypsinogen‐1 and ‐2 escape into the blood and pass through the glomerular filtration membrane of the kidney; however, most trypsinogen‐1 is reabsorbed in the renal tubules. Hence, it is not excreted in the urine, and only trypsinogen‐2, which has a low reabsorption rate, is excreted.

It has been reported that PEP, which is one of the major complications of ERCP, occurs in 5% of diagnostic ERCP cases, 7% of therapeutic ERCP cases, and up to 25% of patients with sphincter Oddi dysfunction after endoscopic balloon dilation or those with a history of PEP.^9^, ^10^, ^11^ Most patients (98%) in this study underwent therapeutic ERCP. The incidence of PEP was 5%. This result is similar to those of previous reports on the incidence of PEP.

In this study, five of 100 patients developed PEP, and all of them had a positive rapid urinary trypsinogen‐2 test after ERCP, with a sensitivity of 100%, specificity of 83.2%, PPV of 23.8%, and NPV of 100%.

Kemppainen et al. tested 106 patients who underwent ERCP using a urinary trypsinogen‐2 test 6 h after ERCP and found that 11 (10.4%) of these patients developed PEP, indicating that the sensitivity and specificity of the rapid urinary trypsinogen‐2 dipstick test were 81% and 97%, respectively.^12^ Additionally, Tseng et al. tested 150 patients who underwent ERCP and obtained measurements with the rapid urinary trypsinogen‐2 dipstick test before ERCP and 3 h after ERCP; they found that 13 (8.7%) of these patients developed PEP, which indicated that the sensitivity and specificity of the rapid urinary trypsinogen‐2 dipstick test 3 h after ERCP were 84.6% and 97.1%, respectively.^13^ Furthermore, Yewale et al. tested 79 patients who underwent ERCP using a urinary trypsinogen‐2 test 4 h after ERCP and found that three (3.8%) of them developed PEP, which indicated that the sensitivity and specificity of the rapid urinary trypsinogen‐2 dipstick test were 66.7% and 92.1%, respectively.^14^ The diagnostic performance of the rapid urinary trypsinogen‐2 dipstick test in the present study was similar to that reported previously.

In addition, the rapid urine trypsinogen‐2 dipstick test can be used even 1 h after ERCP, based on clinical experience, and has a diagnostic performance equivalent to that of serum amylase and serum lipase levels at 8 a.m. of the day after ERCP. Since blood tests are generally performed the day after ERCP (at least 8 h after ERCP), the ability to exclude the possibility of PEP 4 h after ERCP (1.0–4.0 h after ERCP) with the rapid urine trypsinogen‐2 test without physical invasion can shorten hospital stay and eliminate the need for further treatment.

Furthermore, in the present study, we tested with the rapid urinary trypsinogen‐2 dipstick test before ERCP, and we examined patients who were positive with the rapid urinary trypsinogen‐2 dipstick test before ERCP. Eight patients tested positive: four patients had chronic pancreatitis, one patient had autoimmune pancreatitis, one patient had cholangiocarcinoma, one patient had intraductal papillary mucinous neoplasm, and one patient had common bile duct stones. Trypsinogen‐2 is secreted from the biliary epithelium and peribiliary glands, and its levels have also been reported to increase in malignant tumors of the gastrointestinal, pancreas, and biliary tract.^15^, ^16^ The positive rapid urinary trypsinogen‐2 test results before ERCP in this study were thought to be due to these effects. It should be noted that in practice, some patients test positive before ERCP. However, since the diagnostic performance, including pre‐positive, was not different from that of amylase and lipase, testing before ERCP is not necessary in clinical practice (Figure 2).

Overall, 21 patients (21%) who tested positive on the rapid urine trypsinogen‐2 dipstick test after ERCP were compared for amylase and lipase levels. Patients with PEP had significantly higher serum amylase and lipase levels than those without PEP (*p* < 0.001; Figure 3). Thus, even with a positive rapid urine trypsinogen‐2 dipstick test, serum amylase and lipase levels may vary. Moreover, a rapid urine trypsinogen‐2 dipstick test at 4 h (1.0–4.0 h) after ERCP may frequently have a false‐positive result. this limitation of the test should be acknowledged.

One limitation of this study was the small number of PEP cases due to the small sample size of this study, and further investigation is needed in order to estimate the diagnostic accuracy of the rapid urinary trypsinogen‐2 test. A second limitation is that there was a variation in the time of urine collection. In the protocol of the present study, the post‐ERCP rapid urinary trypsinogen‐2 test was conducted 1–4 h after ERCP, and the median time of the test was 4 h (1.0–4.0 h). The reason for the variability in urine collection times was thought to be poor wakefulness due to the use of sedatives (flunitrazepam or dexmedetomidine hydrochloride) and dehydration due to fasting on the day of ERCP. Therefore, using the rapid urine trypsinogen‐2 dipstick test in clinical practice 4 h after ERCP is preferable.

The present study had a small sample size from a single center, therefore, a larger multicenter trial is needed to demonstrate the utility and convenience of this test in routine clinical practice.

## CONCLUSION

The rapid urinary trypsinogen‐2 test could serve as a noninvasive early screening test to exclude the possibility of PEP. The test was performed 1 h after the ERCP. A negative result efficiently excluded the possibility of PEP and also ensured that the next day's serum amylase or lipase levels did not exceed three times the upper reference limit.

## CONFLICT OF INTEREST STATEMENT

Takao Itoi is the Editor‐in‐Chief of *DEN Open*. The rest of the authors declare no conflict of interest.
